# Berberine Toxicity Profile in Experimental Models as a Basis for Assessing Its Biological Safety

**DOI:** 10.3390/molecules31081350

**Published:** 2026-04-20

**Authors:** Anna Karczmarzyk, Danuta Wojcieszyńska, Agnieszka Nowak, Wojciech Smułek, Urszula Guzik

**Affiliations:** 1 Institute of Biology, Biotechnology and Environmental Protection, Faculty of Natural Science, University of Silesia in Katowice, Jagiellońska 28, 40-032 Katowice, Poland; ainakarczmarzyk@gmail.com (A.K.); danuta.wojcieszynska@us.edu.pl (D.W.); agnieszka.a.nowak@us.edu.pl (A.N.); 2 Institute of Chemical Technology and Engineering, Poznan University of Technology, Berdychowo 4, 60-965 Poznan, Poland; wojciech.smulek@put.poznan.pl

**Keywords:** berberine, toxicity, membrane fluidity, bacteria, *Tetrahymena*, algae

## Abstract

Berberine, a natural alkaloid, is a substance widely used in natural medicine. However, there is a significant knowledge gap regarding the potential negative effects of higher environmental concentrations of berberine resulting from its use as a supplement. Therefore, the aim of this study was to assess its toxicity towards microorganisms and organisms from various trophic levels. The results indicate that berberine may influence the reorganization of bacterial membranes, thereby negatively impacting the environmental microbiome. However, oxidative cell damage, a phenomenon commonly described in the literature, was not demonstrated. At the concentrations used, berberine may even have a protective effect. The analysis of toxicity towards *Tetrahymena*, *Selenastrum*, and *Heterocypris* indicated a similar level of berberine toxicity across these organisms, suggesting that the toxic effect is not species-dependent and that the mechanism of toxicity is probably based on universal cellular mechanisms.

## 1. Introduction

Berberine is an organic chemical compound belonging to the isoquinoline alkaloids, found in many plants. Its medicinal properties have long been used in Chinese medicine [1,2]. Berberine is absorbed in the gastrointestinal tract, but due to its low solubility, it is characterized by low absorption and bioavailability below 1% [1]. After absorption, berberine is widely distributed throughout tissues, and it has been detected in the liver, kidneys, lungs, pancreas, heart, brain, and skeletal muscle. The liver plays a major role in berberine metabolism. The main metabolites are berberrubine, thalifencine, demethyleneberberine, and jatrorrhizine with their glucuronide conjugates. Phase I of detoxification involves cytochrome P450 in the liver and intestines [3,4]. In phase II, the resulting intermediates are conjugated with glucuronic acid, sulfuric acid, and methyl groups, forming final metabolites excreted in urine and bile [3,5].

Due to berberine’s high biological activity, it exhibits numerous health-promoting properties. Among these is its anti-inflammatory effect, resulting from its ability to protect the intestinal epithelial barrier and regulate the transcription of proinflammatory cytokines. Berberine also blocks the inflammatory response by activating 5′AMP-initiated kinase in macrophages, which leads to a reduction in the expression of inflammatory mediator genes and nitric oxide synthase. Berberine has been shown to inhibit cyclooxygenase-2 gene activation in colon cancer cells, thereby reducing prostaglandin production and inflammation. Berberine’s anti-inflammatory activity makes it useful for preventing acute pancreatitis. Berberine has also been shown to positively affect carbohydrate metabolism by increasing glucagon-like peptide-1 (GLP-1) levels by stimulating L cells, thereby stimulating pancreatic β-cells to produce insulin [6,7]. Recent studies have shown that berberine can simultaneously inhibit the proliferation and apoptosis of vascular smooth muscle cells, demonstrating its potential for treating atherosclerosis [8,9]. Increasing attention is being paid to its neuroprotective role and use in the treatment of neurodegenerative diseases [1]. However, despite the many positive effects of berberine, it also has negative aspects. It has been shown that higher concentrations of berberine can cause diarrhea, vomiting, and even death in animals [10]. Higher concentrations may also cause the loss of dopaminergic neurons in the substantia nigra and striatum, leading to impaired motor and cognitive functions [3]. Furthermore, it has been shown that berberine can inhibit CYP enzymes, which may be particularly important in combined therapies with other drugs, leading to non-targeted toxicity [11]. The antibacterial properties of berberine are used in therapies combined with antibiotics.

The mechanism of berberine action involves inhibiting DNA replication, RNA transcription, and protein synthesis; influencing enzyme activity; affecting the integrity of cell membranes and cell walls; and influencing cell division and vacuolation [12,13,14]. At the same time, berberine may negatively affect the gut microbiota, leading to dysbiosis [9]. The current literature indicates that despite widespread use of berberine in medicine and the increasing use of its derivatives in adjunctive therapies, its mechanism of action remains complex and multifaceted. Therefore, comprehensive toxicological studies are necessary, including not only the assessment of human safety but also the analysis of impacts on non-target organisms. This allows for a more comprehensive understanding of the potential effects of long-term exposure to this compound.

This is particularly important given the current knowledge gap regarding environmental concentrations of berberine. Until recently, the only environmental source of berberine was plants that produce this compound, such as *Berberis vulgaris*, *Coptis chinensis*, and *Hydrastis canadensis* [1,2]. Therefore, berberine concentrations in soil were relatively low. This is especially true because the compound is subject to transformation and adsorbed by soil colloids. However, the widespread use of concentrated berberine preparations carries the risk of higher concentrations, as it is only slightly metabolized in the body. It can also enter the environment through improper waste separation. Therefore, the environmental risk posed by berberine remains unidentified. This study analyzed its impact on organisms at various trophic levels, enabling an assessment of the potential environmental effects of introducing berberine into ecosystems not previously exposed to it. Moreover, the toxicity of berberine to selected microorganisms was assessed, enabling a broader evaluation of the risks associated with its use in antibacterial therapy.

## 2. Results and Discussion

Despite the rapid expansion of the pharmaceutical market, there is currently a trend toward returning to natural products, as most societies believe they are completely safe. The particularly widespread use of natural products is observed in Asian countries, where this stems from tradition. Therefore, plant-derived commercial products are increasingly common and are sold largely outside strict controls. In turn, people’s belief in the safety of such preparations often leads them to be used at doses higher than recommended. One such product is the alkaloid berberine, obtained from the *Berberis vulgaris* shrub. It is considered an immunomodulator and antibacterial agent, and is also said to have protective effects on the nervous, vascular, and endocrine systems [15]. However, reports of berberine’s toxic effects are increasingly common. Berberine use has been shown to cause damage to the lungs and liver, among other things, and to increase parameters such as alanine and aspartate aminotransferases. Its cytotoxic effects, manifested by disturbances in DNA synthesis, have also been demonstrated. Furthermore, berberine inhibits enzymes such as acetylcholinesterase and monoamine oxidase and may disrupt dopamine biosynthesis [10].

The use of berberine, which is not fully metabolized in the body but is only converted to hydrophilic derivatives, results in its condensed form ending up in wastewater treatment plants and the environment. Due to the real risk of berberine toxicity and its increasing use, it is important to determine the actual threats posed by its use and release into the aquatic environment, where it may affect organisms previously unexposed to its effects.

The negative impact of berberine on microorganisms has so far been described primarily in the context of pathogenic bacteria, with little consideration given to its impact on the human gut microbiome and environmental bacteria, which play a key role in the functioning of ecosystems, including the degradation of pollutants and the circulation of elements in nature. Therapeutic doses of berberine used in antimicrobial therapy for *Helicobacter pylori* infections range up to 1000 mg/day, while minimal inhibitory concentrations (MICs) against this microorganism reach 100 mg/mL [16,17]. The use of such high concentrations may result in the release of berberine into the environment at levels that harm naturally occurring organisms. This risk may be further increased by the photosensitizing properties of berberine—in the presence of this compound, under the influence of UV radiation, singlet oxygen and other reactive oxygen species are produced, which can lead to DNA damage and a reduction in cell viability by up to 80%, which significantly increases the risk associated with the presence of berberine in wastewater [18].

**Figure 1:**
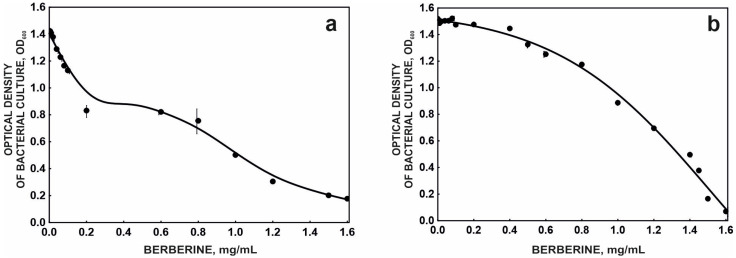
Growth inhibition curve of *Escherichia coli* DH5α (**a**) and *Pseudomonas moorei* KB4 (**b**) in the presence of berberine at various concentrations.

In light of these risks, this study assessed the effect of berberine on two bacterial strains of different origin and ecological significance. The *Pseudomonas moorei* KB4 strain is an environmental strain characterized by enhanced degradation of paracetamol and diclofenac. The second strain studied, *Escherichia coli* DH5α, served as a control strain because it is a laboratory strain. Both strains were exposed to berberine at concentrations ranging from 0 to 1.6 mg/mL. The DH5α strain was more sensitive to berberine’s toxic effects than the KB4 strain (Figure 1). The EC_50_ values were 0.89 mg/mL and 1.16 mg/mL for the DH5α and KB4 strains, respectively.

**Figure 2:**
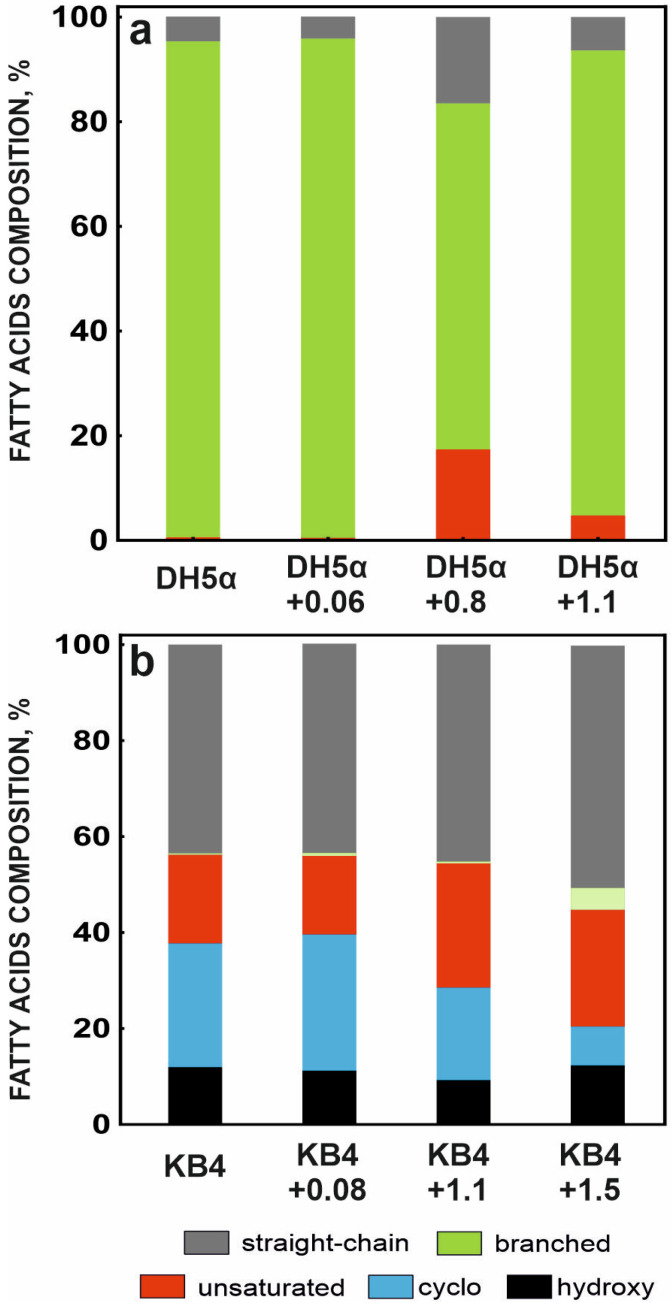
Percentage of total fatty acids in *Escherichia coli* DH5α (**a**) and *Pseudomonas moorei* KB4 (**b**) after berberine (mg/mL) exposure. The data are presented as the mean values of three biological replicates.

Analysis of fatty acids isolated from cells of the studied strains revealed dramatic differences in their profiles. Strain DH5α showed a very high proportion of branched-chain fatty acids, both in cells exposed to berberine and in those cultured under control conditions. In strain KB4, however, small amounts of this fatty acid group were observed only in cells exposed to the highest (1.5 mg/mL) concentration of berberine. The dominant type of fatty acids in this strain was straight-chain fatty acids. Such differences indicate differences in cell membrane fluidity. The cell membrane of strain DH5α exhibits greater fluidity, allowing berberine to intercalate into its structure. This may lead to changes in permeability, thereby disrupting cellular metabolic processes. In response to the highest concentration (1.2 mg/mL), which caused significant growth inhibition, the strain synthesized unsaturated fatty acids (Figure 2a, Table 1).

This was likely a response to excessive membrane stiffening resulting from berberine’s incorporation into the lipid bilayer’s loosened structure. In turn, exposure to higher concentrations of berberine from strain KB4 resulted in an increased proportion of unsaturated fatty acids and a decreased proportion of cyclopropane acids (Figure 2b, Table 2).

The more ordered structure of the KB4 strain’s cell membrane may hinder berberine’s incorporation, thereby protecting cells from its harmful effects. Berberine’s negative effect on membrane structure is likely related to changes in membrane permeability. It should be emphasized that the results of this study do not confirm reports by other authors of cell membrane loosening under the influence of berberine and the accompanying efflux of cellular components [16]. Analysis of changes in the fatty acid composition of the tested strains after exposure to berberine indicates that the observed modifications represent a cellular response to excessive membrane stiffening (Figure 2, Table 1 and Table 2). This suggests that berberine’s action may increase membrane stiffness, leading to an effect similar to that of steroid compounds. Because of the lack of extensive hydrogen bonding, berberine, in its positively charged form, can incorporate into lipid bilayers. It can also penetrate into the cell and facilitate proton transport across membranes, react with redox groups of the respiratory chain, induce DNA damage, and inhibit enzymes [19].

The literature suggests that membrane damage may be oxidative in nature [15,16]. However, analysis of the main marker of lipid peroxidation—malondialdehyde—revealed that in the presence of berberine, a significant decrease in the level of this intermediate was observed in the DH5α strain. Moreover, no significant changes were observed in the KB4 strain (Table 3). These results suggest that berberine does not induce fatty acid oxidation and may even have a protective effect. This is also confirmed by reduced catalase levels in the DH5α strain and only slightly increased superoxide dismutase activity. Based on this, it can be inferred that in the presence of berberine, oxidative damage to membrane lipids does not occur in either of the tested strains. The toxic effects of berberine are most likely due to its direct effect on cell membrane permeability [20,21].

The effect of berberine on the DH5α strain was also assessed based on phosphatase activity. Following cell exposure to berberine, a significant increase in both acid and alkaline phosphatase activity was observed (Table 3). Although the increase in the activity of these enzymes is often interpreted as an indicator of cellular damage, including membrane destabilization and disturbances in phosphorus metabolism, the results of this study do not support this mechanism [22].

In particular, no evidence of membrane structural loosening or oxidative damage was observed (Table 3). On the contrary, a greater proportion of saturated fatty acids was observed, which increased membrane stiffness and, consequently, decreased membrane permeability (Figure 2), indicating that berberine does not lead to membrane disintegration but rather decreases membrane fluidity and increases lipid ordering.

The simultaneous increase in phosphatase activity and increased membrane stiffness should therefore be interpreted as a secondary, adaptive response of cells to changes in the physicochemical properties of the membrane. Berberine’s interaction with phospholipids can stiffen the lipid bilayer, limiting transport across the membrane. In response to this change, cells activate enzymes involved in phospholipid and phosphate metabolism, including phosphatases, which participate in membrane remodeling and maintenance of cellular homeostasis.

Based on the obtained results, a model of berberine’s action can be proposed: the compound interacts with membrane lipids, increasing their order and decreasing membrane fluidity, thereby reducing permeability. These changes initiate a secondary adaptive cellular response, including the activation of enzymes involved in phosphorus and phospholipid metabolism, which help maintain membrane integrity and functionality under berberine-induced stress conditions. Our hypothesis is based primarily on correlations and requires further experimental studies to confirm a direct mechanism of berberine’s action on the cell membrane. The main limitations of this hypothesis include the lack of direct measurements of membrane fluidity and spectroscopic studies of berberine-cell membrane interactions. Furthermore, increased phosphatase activity may result not only from membrane remodeling but also from a cellular stress response, altered phosphate availability, or metabolic disturbances unrelated to the membrane. Although oxidative stress was not observed in the studied bacteria following berberine exposure, numerous reports in the literature suggest that the primary mechanism of action of berberine is increased production of reactive oxygen species. This likely results from its effect on the first complex of the respiratory chain, which is inhibited by high berberine concentrations [23]. Therefore, it was important to examine how berberine might affect eukaryotic organisms with a typical respiratory chain structure. The study included organisms from various trophic levels: algae, protozoa, and ostracods. It demonstrated that all of these organisms, despite significant taxonomic differences and varying levels of cellular organization, exhibit similar sensitivity to berberine. This may suggest that the toxic effects of this compound result from its impact on universal cellular structures and processes, such as the cell membrane, energy metabolism, or mechanisms regulating oxidative balance [15,16].

The complexity of berberine’s effects is also indicated by numerous scientific reports demonstrating its ability to reduce oxidative stress in cells. This was argued to be due to its influence on cellular signaling pathways, particularly those related to the Nrf2 pathway, either through inhibition of sirtuin-1-mediated p66Shc signaling, which has been implicated in modulating reactive oxygen species, or through activation of peroxisome proliferator-activated receptor gamma (PPARc), a transcription factor that counteracts oxidative stress and inflammation, leading to increased expression of endogenous antioxidant defense mechanisms [24,25]. Bacterial cells, on the other hand, lack homologous regulatory pathways and exhibit distinct mechanisms of redox homeostasis. As a result, antioxidant effects observed in eukaryotic models may not translate directly to bacterial systems. It is also important to consider that berberine’s effects on oxidative balance may depend on factors such as concentration, exposure time, and bacterial strain. In some cases, berberine has been reported to induce oxidative stress rather than alleviate it, suggesting a potential dual or hormetic mode of action. Therefore, the lack of observed antioxidant effects in this study does not contradict existing literature but rather highlights the complexity and context-specific nature of berberine’s biological activity.

**Figure 3:**
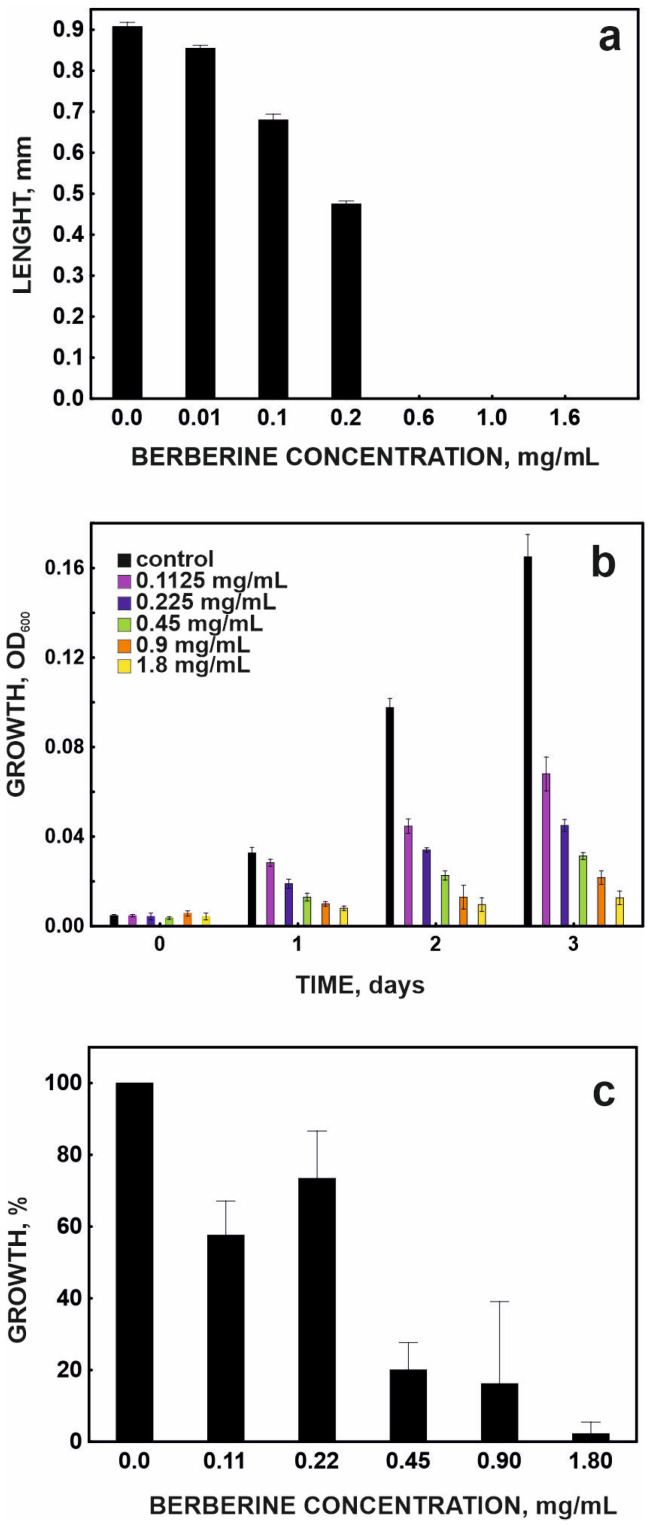
Effect of berberine on the growth of *Heterocypris incongruens* (**a**), *Selenastrum capricornutum* (**b**), and *Tetrahymena thermophila* (**c**).

It was observed that the effect of berberine on planktonic *Selenastrum* cells depended not only on the dose but also on the exposure time. The EC_50_ of berberine determined after 24 h of exposure was 0.21 mg/mL for this alga, while after 48 and 72 h it was 0.09 and 0.08 mg/mL, respectively. This indicates that the effect of berberine is cumulative, and the timing of exposure is an important factor in determining its toxicity. This phenomenon may result from the gradual accumulation of the compound in cells, disruption of cell membrane function, or intensification of oxidative stress during long-term exposure [10]. The EC_50_ of berberine was determined to be 0.35 and 0.2 mg/mL for *Tetrahymena* and *Ostracod*, respectively. These values indicate that although these organisms belong to different taxonomic groups and differ significantly in cellular organization, they respond similarly to higher berberine concentrations (Figure 3).

The models’ sensitivity to berberine does not clearly indicate a common mechanism of toxicity, despite the similar endpoint. The mechanism of berberine toxicity is complex, acting at multiple levels of cellular organization and affecting fundamental cellular processes common to many eukaryotic organisms. The lethal effect in these organisms may probably result not only from the inhibition of complex I of the respiratory chain and the resulting high oxidative stress, but also from the influence of berberine on the synthesis of nucleic acids, as this compound may directly interact with DNA or act as a topoisomerase inhibitor [23,26,27].

From an ecological perspective, these results are particularly significant because they indicate that berberine can affect organisms across various trophic levels within planktonic communities. Consequently, its presence in aquatic environments could influence the structure and functioning of food webs, as well as primary production and material transfer in these ecosystems.

## 3. Materials and Methods

### 3.1. Bacterial Strains and Chemicals

The study used the environmental strain *Pseudomonas moorei* KB4, isolated from activated sludge at the Klimzowiec wastewater treatment plant (Chorzów, Poland) [28], and the reference strain *Escherichia coli* DH5.

The chemicals employed in this study were of the highest purity grade. The berberine used in the research was purchased from Merck (technical purity ≥ 90%).

### 3.2. Bacterial Growth Inhibition Test

The effect of berberine on bacterial growth was assessed using pure cultures of *Pseudomonas moorei* KB4 and *Escherichia coli* DH5α. The strains were grown in LB medium supplemented with increasing concentrations of berberine (0–1.6 mg/mL). Each culture was standardized to an initial optical density (OD_600_) of 0.1. After 24 h of incubation at 30 °C under continuous shaking, bacterial growth was quantified by measuring the optical density at 600 nm.

### 3.3. Toxicity Bioassays

To assess the chronic toxicity of berberine, Ostracodtoxkit F, Protoxkit F, and Algaltoxkit tests were performed according to the manufacturer’s instructions (TIGRET Sp. z o.o., Warsaw, Poland). The concentration range of berberine used in the Ostracodtoxkit F test with *Heterocypris incongruens* was 0.01–1.6 mg/mL. In the Protoxkit F test with *Tetrahymena thermophila* and in the Algaltoxkit test with *Selenastrum capricornutum*, berberine was tested at concentrations ranging from 0.1125 to 1.8 mg/mL.

### 3.4. Fatty Acids Extraction and Analysis

The fatty acid profiles of the examined strains were examined after 24 h of cultivation under two experimental conditions: (i) nutrient broth without additives (control), and (ii) nutrient broth supplemented with berberine. For strain KB4, fatty acid profiles were analyzed after exposure to concentrations of 0.08 mg/mL, 1.1 mg/mL, and 1.5 mg/mL berberine. For strain DH5α, fatty acid profiles were analyzed after exposure to concentrations of 0.06 mg/mL, 0.8 mg/mL, and 1.2 mg/mL berberine.

Bacterial biomass was harvested by centrifugation at 8000× *g* for 20 min at 4 °C. The obtained cell pellets were subsequently rinsed twice with sterile 0.85% NaCl solution to remove residual components of the growth medium.

Fatty acid extraction and identification were carried out using the MIDI Microbial Identification System (MIDI-MIS) in accordance with the procedure described by Żur et al. [28]. Fatty acid methyl esters (FAMEs) were analyzed with an HP 5890 gas chromatograph (Hewlett Packard, Rolling Meadows, IL, USA) fitted with a 25 m × 0.2 mm capillary column coated with cross-linked methyl silicone. The temperature program was initiated at 170 °C, then increased to 260 °C at 5 °C/min, followed by a rapid rise to 320 °C at 40 °C/min, with a final hold of 1.5 min. Helium was used as the carrier gas during the chromatographic runs. FAMEs were identified using Sherlock software (TSBA library, version 3.9, Microbial ID, Newark, NJ, USA) based on previously calibrated retention times reported by Żur et al. [28].

The average fatty acid chain length (Mean FA) was determined according to the formula described by Yang et al. [29]. All statistical analyses were performed with the Statistica 13.0 PL software package. One-way analysis of variance (ANOVA) was used to assess differences between control and treated samples, with *p* < 0.05 set as the threshold for statistical significance.

### 3.5. Preparation of Cell Extracts

Cell-free extracts were obtained from isolated bacteria harvested during the exponential growth phase (OD600 < 0.8). Crude enzyme preparations were obtained from cultures grown in LB medium with berberine (0.6 mg/mL) or in LB medium as a control. Cells were collected by centrifugation at 4500× *g* for 15 min at 4 °C, followed by washing with 50 mM phosphate buffer (pH 7.0). The pellets were subsequently resuspended in the same buffer and disrupted by sonication (six cycles of 15 s each). Cell debris was removed by centrifugation at 9000× *g* for 30 min at 4 °C. The resulting clarified supernatants were used as crude cell-free extracts in enzymatic activity assays [30].

### 3.6. Enzyme Assays

Catalase activity was estimated by measuring the decrease in absorbance at 240 nm with 54 mM H_2_O_2_ [30]. The activity of superoxide dismutase was measured using the SOD Assay Kit (Sigma-Aldrich, St. Louis, MO, USA) according to the manufacturer’s instructions. One unit (U) of the enzyme activity was defined as the amount of enzyme required to generate 1 µmol of product per minute.

The activities of alkaline and acid phosphatases were determined by measuring the formation of p-nitrophenol at 415 nm in both crude extracts. The activity of alkaline and acid phosphatase was calculated in Bessey units, denoting the amount of mmol of *p*-nitrophenol released by the enzyme in 1000 mL of the enzyme fraction during 1 h of incubation [30].

Protein concentration in the crude extract was determined by the Bradford method using bovine serum albumin as a standard [30].

### 3.7. The Lipids Peroxidation Assay

Lipid peroxidation was assessed by mixing 1 mL of crude cell extract with 1 mL of trichloroacetic acid solution (15%, *w*/*v*, prepared in 0.25 M HCl) and 1 mL of thiobarbituric acid solution (0.37%, *w*/*v*, in 0.25 M HCl). The reaction mixtures were heated at 100 °C for 10 min and subsequently allowed to cool to room temperature. After cooling, the samples were centrifuged at 5000× *g* for 20 min at 4 °C. The absorbance of the resulting supernatants was determined spectrophotometrically at 535 nm. Two blank controls were included: one in which distilled water replaced the crude extract, and another in which water replaced the thiobarbituric acid reagent [31]. The levels of lipid peroxidation products were calculated using the molar extinction coefficient of malondialdehyde (ε = 1.56 × 10^5^ M^−1^ cm^−1^).

## 4. Conclusions

The results indicate that berberine has a significant impact on environmental organisms, particularly at high concentrations. Because it occurs naturally in plants, this compound does not pose a significant environmental threat under natural conditions. However, it should be noted that the increasing production of dietary supplements containing high doses of berberine and their long-term use in therapies may lead to increased release of this alkaloid into the environment. Consequently, it may accumulate in aquatic ecosystems at concentrations potentially harmful to aquatic organisms. Therefore, it seems justified to monitor the presence of berberine in the environment and introduce appropriate regulations for the dietary supplement market, particularly regarding the control of this compound’s content and compliance with recommended doses.

## Figures and Tables

**Table 1:** Percentage of total fatty acids in *Escherichia coli* DH5α growing in the control systems and in the presence of 0.06, 0.8, and 1.2 mg/mL berberine.

Fatty Acids	% of Total Fatty Acids			
	*Escherichia coli* DH5α	*Escherichia coli* DH5α + 0.06 mg/mL Berberine	*Escherichia coli* DH5α + 0.8 mg/mL Berberine	*Escherichia coli* DH5α + 1.2 mg/mL Berberine
Saturated				
11:0 *iso*	0.34 ± 0.07	0.22 ± 0.25	0.00 ± 0.00	0.00 ± 0.00
11:0 *anteiso*	0.19 ± 0.17	0.00 ± 0.00	0.00 ± 0.00	0.00 ± 0.00
12:0	0.00 ± 0.00	0.12 ± 0.09	2.01 ± 0.02	0.00 ± 0.00
12:0 *iso*	0.05 ± 0.09	0.00 ± 0.00	0.00 ± 0.00	0.00 ± 0.00
13:0 *iso*	11.86 ± 1.25	9.99 ± 1.93	8.35 ± 2.06	19.80 ± 0.53
13:0 *anteiso*	9.31 ± 1.14	7.57 ± 1.94	5.92 ± 0.68	11.99 ± 0.56
14:0	0.93 ± 0.06	0.69 ± 0.10	2.40 ± 0.35	2.03 ± 0.50
14:0 *iso*	0.38 ± 0.05	0.39 ± 0.06	0.00 ± 0.00	0.00 ± 0.00
15:0 *iso*	16.26 ± 0.57	17.38 ± 0.64	13.02 ± 0.79	15.47 ± 1.00
15:0 *anteiso*	37.85 ± 1.55	39.09 ± 1.99	25.25 ± 0.41	28.04 ± 3.28
16:0	1.26 ± 0.05	1.25 ± 0.08	9.71 ± 0.92	4.39 ± 1.41
16:0 *iso*	0.62 ± 0.05	0.51 ± 0.02	0.00 ± 0.00	0.00 ± 0.00
17:0 *iso*	7.94 ± 0.51	9.30 ± 0.67	6.42 ± 0.07	6.95 ± 0.44
17:0 *anteiso*	6.90 ± 0.39	7.25 ± 0.39	5.12 ± 0.14	5.47 ± 0.38
18:0	1.72 ± 0.16	1.57 ± 0.22	2.38 ± 0.00	0.00 ± 0.00
19:0 *iso*	1.92 ± 0.17	2.50 ± 0.42	2.05 ± 0.15	1.08 ± 1.52
19:0 *anteiso*	1.03 ± 0.07	1.19 ± 0.09	0.00 ± 0.00	0.00 ± 0.00
20:0	0.54 ± 0.16	0.57 ± 0.04	0.00 ± 0.00	0.00 ± 0.00
Unsaturated				
16:1 ω7c/16:1 ω6c	0.00 ± 0.00	0.46 ± 0.10	10.19 ± 0.56	2.99 ± 1.52
18:1 ω7c	0.00 ± 0.00	0.00 ± 0.00	7.17 ± 0.44	1.79 ± 2.52
18:1 ω9c	0.58 ± 0.67	0.00 ± 0.00	0.00 ± 0.00	0.00 ± 0.00
Mean Lenght	14.09 ± 1.39	13.98 ± 0.08	15.24 ± 0.07	14.48 ± 0.06
Sat./unsat. ratio	172.06 ± 1.37	221.70 ± 48.06	4.78 ± 0.33	31.73 ± 27.75

ω—methyl end of fatty acid. *c*—*cis* configuration of the double bond. *iso*, *anteiso*—branched fatty acids.

**Table 2:** Percentage of total fatty acids in *Pseudomonas moorei* KB4 growing in the control systems and in the presence of 0.08, 1.1, and 1.5 mg/mL berberine.

Fatty Acids	% of Total Fatty Acids			
	*Pseudomonas moorei* KB4	*Pseudomonas moorei* KB4+ 0.08 mg/mL Berberine	*Pseudomonas moorei* KB4 + 1.1 mg/mL Berberine	*Pseudomonas moorei* KB4 + 1.5 mg/mL Berberine
Saturated				
10:0	0.11 ± 0.16	0.18 ± 0.01	0.00 ± 0.00	0.44 ± 0.26
10:0 3OH	3.94 ± 0.12	3.70 ± 0.07	2.55 ± 0.32	3.46 ± 0.53
12:0	4.42 ± 0.01	4.11 ± 0.03	4.40 ± 0.41	4.74 ± 0.45
12:0 2OH	3.35 ± 0.06	3.22 ± 0.04	2.98 ± 0.15	3.72 ± 0.20
12:0 3OH	4.64 ± 0.05	4.29 ± 0.11	3.70 ± 0.35	5.13 ± 0.18
14:0	1.37 ± 0.01	1.45 ± 0.05	2.11 ± 0.16	1.68 ± 0.06
14:0 3OH	0.00 ± 0.00	0.00 ± 0.00	0.47 ± 0.81	0.00 ± 0.00
16:0	37.07 ± 0.16	37.35 ± 0.39	37.57 ± 1.30	42.66 ± 0.78
17:0 *iso*	0.00 ± 0.00	0.00 ± 0.00	0.00 ± 0.00	0.71 ± 0.00
17:0 *cyclo*	25.22 ± 0.01	27.13 ± 0.21	19.27 ± 0.99	7.25 ± 0.87
18:0	0.52 ± 0.04	0.55 ± 0.01	1.11 ± 1.06	0.96 ± 0.15
19:0 *iso*	0.34 ± 0.47	0.63 ± 0.06	0.00 ± 0.00	4.58 ± 0.27
19:0 *cyclo* ω9c	0.57 ± 0.81	1.25 ± 0.03	0.00 ± 0.00	0.84 ± 0.15
Unsaturated				
16:1 ω7c/16:1 ω6c	12.06 ± 0.64	10.44 ± 0.17	15.46 ± 0.40	13.62 ± 1.85
17:1 ω7c	0.00 ± 0.00	0.00 ± 0.00	0.00 ± 0.00	1.27 ± 0.17
18:1 ω7c	6.39 ± 0.24	5.91 ± 0.07	10.38 ± 0.94	9.42 ± 0.79
Mean Lenght	15.65 ± 0.02	15.72 ± 0.02	15.77 ± 0.06	15.61 ± 0.05
Sat./unsat. ratio	4.43 ± 0.26	5.11 ± 0.07	2.87 ± 0.13	3.14 ± 0.45

ω—methyl end of fatty acid. *c*—*cis* configuration of the double bond. *iso*—branched fatty acids.

**Table 3:** Level of oxidative stress markers in *Escherichia coli* DH5α and *Pseudomonas moorei* KB4 cells in the control systems and after exposure to 0.6 mg/mL berberine.

Marker	*Escherichia coli* DH5α	*Escherichia coli* DH5α + Berberine	*Pseudomonas moorei* KB4	*Pseudomonas moorei* KB4 + Berberine
Malondialdehyde	0.20 ± 0.02 μM	0.09 ± 0.03 μM	0.44 ± 0.03 μM	0.42 ± 0.03 μM
Catalase	29.20 ± 0.92 U/mg of protein	21.78 ± 0.24 U/mg of protein	4.36 ± 0.05 U/mg of protein	5.89 ± 0.19 U/mg of protein
Dismutase	3.46 ± 0.60 U/mg of protein	4.25 ± 0.06 U/mg of protein	1.51 ± 0.09 U/mg of protein	1.65 ± 0.03 U/mg of protein
Acid phosphatase	6.72 ± 0.58 Bessey units/mg of protein	24.62 ± 2.73 Bessey units/mg of protein	4.83 ± 0.05 Bessey units/mg of protein	2.70 ± 0.07 Bessey units/mg of protein
Alkaline phosphatase	7.06 ± 0.47 Bessey units/mg of protein	28.13 ± 2.30 Bessey units/mg of protein	0.31 ± 0.06 Bessey units/mg of protein	3.09 ± 0.32 Bessey units/mg of protein

## Data Availability

The data presented in this study are available on request from the corresponding author.
